# The effect of oral zonisamide treatment on serum phenobarbital concentrations in epileptic dogs

**DOI:** 10.3389/fvets.2024.1389615

**Published:** 2024-05-29

**Authors:** Elizabeth Mahon, Oliver Marsh, Ane Uriarte, Fabio Stabile

**Affiliations:** ^1^Department of Neurology and Neurosurgery, Southfields Veterinary Specialists, Part of Linnaeus Ltd, Essex, United Kingdom; ^2^Department of Neurology and Neurosurgery, Dick White Referrals, Part of Linnaeus Ltd, Newmarket, United Kingdom; ^3^Department of Neurology and Neurosurgery, Wear Referrals, Part of Linnaeus Ltd, Bradbury, United Kingdom

**Keywords:** zonisamide, phenobarbital, epilepsy, dogs, idiopathic epilepsy, structural epilepsy

## Abstract

Zonisamide is used in dogs for the treatment of epileptic seizures. It is predominantly metabolised by CYP450 hepatic enzymes. When used concurrently with phenobarbital (PB), zonisamide clearance is increased and its elimination half-life decreases. However, the effect that zonisamide may have on serum PB concentrations in dogs has not been previously described. Eight dogs diagnosed with idiopathic epilepsy and two dogs with structural epilepsy commenced zonisamide at 8.0 mg/kg/12 h [7.4–10 mg/kg/12 h] following an increase in the frequency of epileptic seizures. Nine dogs were receiving PB every 12 h (4.2 mg/kg/12 h [3.8–6 mg/kg/12 h]), and one dog was receiving PB every 8 h (6 mg/kg/8 h). Following the addition of zonisamide and despite no increase in PB dosage, an increase in phenobarbital serum PB concentration was observed in 9 out of 10 dogs in subsequent measurements. In five dogs, phenobarbital serum concentrations were raised to concentrations higher than the reported hepatotoxic concentrations (trough>35 mg/L). This required a reduction in daily doses of PB. This case series suggests that zonisamide affects the metabolism of PB and causes an increase in PB serum concentrations over time.

## Introduction

Epilepsy is a disease characterised by a predisposition to generate epileptic seizures (ES) (1). Reactive seizures occur due to transient disturbance in brain function from metabolic or toxic causes. Epileptic seizures can be caused by idiopathic or structural epilepsy. Structural epilepsy (StrE) is caused by intracranial pathology, for example vascular, inflammatory/infectious, traumatic, developmental, neoplastic, and degenerative disease (1). Idiopathic epilepsy (IE) is a diagnosis of exclusion based upon the age of ES onset (usually 6 months to 6 years), unremarkable biochemistry, complete blood cell count (CBC) and urinalysis, and normal interictal general and neurological examinations. Diagnosis can be further supported by normal bile acid stimulation testing, magnetic resonance imaging (MRI) of the brain, cerebrospinal fluid (CSF) analysis, and electroencephalography. A tier I–III confidence level for the diagnosis of IE can then be given, depending on which criteria are met (1, 2).

Phenobarbital (PB) is the most common first-line anti-seizure drug (ASD) used in veterinary medicine for the treatment of epilepsy, with a reported efficacy of 60–93% in dogs with IE (3, 4). However, an additional ASD should be commenced when there is unsatisfactory ES control despite the serum PB concentration (sPBc) being at the top end of the recommended reference interval (15–35 mg/L) (5, 6).

Zonisamide (ZN) is an ASD used both as a monotherapy and adjunctive therapy in dogs with IE (7–9). It is mostly used as a second- or third-line ASD in Europe and the United States and as a first-line ASD in Japan (5, 10). The mechanism of action of ZN is not completely understood but is believed to mainly involve the inhibition of voltage-gated sodium channels and T-type calcium channels in excitatory neurons. It may also enhance gamma-aminobutyric acid (GABA) release, inhibit glutamate release, scavenge free radicals, and increase dopamine and serotonin synthesis (3, 5, 11).

PB is metabolised by hepatic enzymes and is a potent inducer of cytochrome P450 enzymes, including CYP2C and CYP3A (5, 12, 13). Chronic administration of PB in dogs increases its own clearance and decreases its own elimination half-life, which can result in a decrease in sPBc over time (3, 14). Zonisamide is predominantly metabolised by CYP2C and CYP3A hepatic enzymes in humans (15). It has been shown that the elimination half-life, bioavailability, and maximum serum concentrations of ZN are decreased when given concurrently with target dosages of PB (12). Therefore, the recommended starting dosage of ZN is increased from 3–7 mg/kg/12 h to 7–10 mg/kg/12 h when given concurrently with PB in epileptic dogs (3).

Although the effect of PB on the pharmacokinetics of ZN has been analysed in previous studies, there are no reports about the effects of ZN on sPBc in dogs. The present study aimed to evaluate the sPBc in dogs with epilepsy following the initiation of ZN as a second- or third-line ASD and any adverse effects of the treatment combination.

## Materials and methods

The medical records of dogs treated for epilepsy at a single institution (Southfields Veterinary Specialists, Linnaeus Ltd), between August 2020 and August 2023, were retrospectively reviewed to assess the effect of oral ZN treatment on sPBc.

All data were reviewed to match the following inclusion criteria: All dogs must have had a complete physical and neurological examination by a board-certified neurologist and were diagnosed with IE or StrE based on investigations, including CBC, serum biochemistry, and electrolyte analysis, bile acid stimulation test, urinalysis, magnetic resonance imaging (MRI) of the brain, and cerebrospinal fluid (CSF) analysis. Dogs with IE must have met the criteria for diagnosis based on a tier II confidence level as per Intentional Veterinary Epilepsy Task Force (IVETF) recommendations (2). Dogs must have been receiving both PB and ZN to be included in the study. Dogs must have had sPBc measured before starting on and while receiving oral ZN and then subsequently had sPBc measured after at least 3 weeks when the dosage of PB had not been increased. Peak sPBcs were measured 4 h after oral PB administration, and trough sPBcs were measured just before the next oral dosage, either 8 or 12 h later (5). The sPBcs and sZNcs were measured at least two weeks after a dosage change, if epileptic seizure frequency increased or as part of routine monitoring (5).

The following data were collected: age, breed, body weight, type of epilepsy, dose and frequency of administration of ASDs, serum concentrations of ASDs, formulation of PB medication, side effects of ASDs, and the results of serum triglyceride concentration and bile acid stimulation tests (BAST) if taken. The results of BAST were used as an assessment of liver function (5, 16). Serum triglyceride concentrations >11.3 mmol/L have been associated with a false elevation in sPBc and were therefore collected to assess if our results could be impacted by an elevation in serum triglyceride (17). The referenced target range of PB was 15–35 mg/L and for ZN was 10–40 mg/L (5). The PB elimination half-life was calculated according to previous publications (18). The sPBcs were measured with Immulite 2000 XPi ® (Siemens). The sZNcs were measured by Agilent © 6460 mass spectrometer.

## Results

### Epileptic seizure aetiology

Ten dogs met the inclusion criteria (Table 1). Eight out of ten dogs were diagnosed with IE with Tier II confidence according to the IVETF guidelines (2) and 2 out of 10 dogs were diagnosed with StrE. One of two dogs with StrE (dog 7) was diagnosed with an intra-axial mass lesion affecting the left piriform and frontal lobes, suspected to be neoplastic in origin based on MRI features and normal CSF analysis (19). One of two dogs with StrE (dog 5) was diagnosed with multifocal intra-axial haemorrhagic lesions within both cerebral hemispheres, predominantly in the left and right parietal and occipital lobes (20). No underlying cause for the intra-axial haemorrhage lesions was found despite extensive investigations including biochemistry, CBC urinalysis, coagulation profile, *Angiostrongylus vasorum* antigen testing, blood pressure monitoring, thoracic radiographs, and abdominal ultrasound.

**Table 1:** Serum phenobarbital concentration (sPBc) before and after the administrations of zonisamide (ZN) and the serum ZN concentration (sZBc).

	Signalment	Diagnosis	PB dosage	ZN dosage	sPBc before ZN started (mg/L)	Time receiving ZN (weeks)	sPBc after ZN started (mg/L)	sZNc (mg/L)
Dog 1	4 years, MN, Crossbreed	IE	6 mg/kg/12 h	10 mg//kg/12 h	P: 34.6 T: 28.1 HL: 26.64	6	**P: 45.0** **T: 37.1** HL: 28.72	20
Dog 2	8 years, MN, Italian Spinone	IE	5 mg/kg/12 h	10 mg/kg/12 h	P: 37.5 T: 31.5 HL: 31.8	3	**P: 44.8** **T: 39.3** HL: 42.33	35
Dog 3	2 years, FN, French bulldog	IE	3.8 mg/kg/12 h	8.3 mg/kg/12 h	P:30.2 T: 25.1 HL: 29.97	2.5	**R: 34.6**	NA
			3.8 mg/kg/12 h	8.3 mg/kg/12 h	NA	8	P: 32.5 T: 29.2 HL: 51.78	21
Dog 4	4 years, FN, French bulldog	IE	6 mg/kg/12 h	10 mg/kg/12 h	P: 32.4 T: 28.1 HL: 38	8	P: 31.3 T: 24.8 HL HL: 34	12
			6 mg/kg/12 h	15 mg/kg/12 h	NA	37	**R: 34.8**	30
			6 mg/kg/12 h	20 mg/kg/12 h	NA	58	**R: 37.3**	35
Dog 5	10 years, MN, Corgi	SE	3 mg/kg/12 h	7.5 mg/kg/12 h	R: 21.6	2.5	Dosage of PB increased and ZN started before sPBc measured^a^	NA
Dog 6	4 years, MN, cross breed	IE	5.2 mg/kg/8 h	10 mg/kg/12 h	P: 41.3 T: 39.6 HL: 65	NA	Dosage of PB decreased and ZN started before sPBc measured^a^	NA
Dog 7	7 years, FN, French bulldog	SE	5.2 mg/kg/12 h	9 mg/kg/12 h	R: 19.0	NA	Dosage of PB increased and ZN started before sPBc measured^a^	NA
Dog 8	1 year, FE, boxer,	IE	3.4 mg/kg/12 h	10 mg/kg/12 h	6hpp: 29.0	4	**6hpp: 34.0**	29
Dog 9	1 year, MN, boxer	IE	4.2 mg/kg/12 h	7.4 mg/kg/12 h	P:32.2 T: 31.8 HL: 443.51	4	P: 32.3 T: 29.1 HL: 53.14	11
Dog 10	5 years, MN, cross breed	IE	4.5 mg/kg/12 h	10 mg/kg/12 h	R: 21.0	NA	Dosage of PB increased and ZN started before sPBc measured^a^	NA

The values in bold represent increases in sPBc following the administrations of ZN and despite no change in PB dosage. P, peak sPBc; T, trough sPBc; R, unknown time of day sPBc taken; 6hpp, 6 h after dose of PB; ME, male entire; MN, male neutered; FE, female entire; FN, female neutered; HL, PB elimination half-life.

a Further details of these cases can be found in Table 2.

### Treatment with antiseizure drugs

All dogs were initially started on PB following epileptic seizure activity and an interictal period of less than 6 months (3). The mean PB starting dosage was 2.5 mg/kg/12 h [1.5–3 mg/kg/12 h]. The oral PB dosage was gradually increased based upon monitoring sPBc and ES control. The information on phenobarbital formulation was available for 7 out of 10 dogs (dogs 2, 4, 5, 7, 8, 9 and 10). Of these seven dogs, six received 30 mg or 60 mg tablets of Epiphen® (Vetoquinol UK Limited) and one received 60 mg tablets of Soliphen (TVM Animal Health Limited). The brand was not changed between sPBc measurements according to treatment history.

In nine out of ten dogs, ZN was started after a mean of 49 weeks [8–125 weeks] after the initiation of PB treatment. In one out of ten dogs (dog 2), it was unclear from the records how long PB was being administered before commencing ZN but it was administered for at least 1 year. When ZN was started, out of ten dogs, nine were receiving PB every 12 h at the mean dosage of 4.2 mg/kg [3.8–6 mg/kg/12 h]) and one was receiving PB every 8 h (6 mg/kg/8 h) (18). The mean sPBc was 25.6 mg/L [21–39.6 mg/L] prior to starting ZN treatment. The mean starting dosage of ZN was 8.0 mg/kg/12 h [7.4–10 mg/kg/12 h].

Additionally, out of 10 dogs, seven (dogs 1, 3, 4, 5, 7, 9, and 10) were receiving levetiracetam (LEV) at a mean dosage of 32 mg/kg/8 h [25–57 mg/kg/8 h], and two (dogs 2 and 6) were receiving both LEV at 30 mg/kg/8 h and 50 mg/kg/8 h, respectively, and potassium bromide (KBR) at 15 mg/kg/48 h and 48 mg/kg/24 h, respectively. Dogs had been receiving LEV for a mean of 3 months (0–6 months) and KBR for 4 months (dog 2) and 10 months (dog 6) before starting ZN.

Therefore, ZN was the second-line ASD in one out of ten dogs, third-line ASD in seven of ten dogs, and fourth-line ASD in two of ten dogs. Dog 7 was also receiving prednisolone (0.3 mg/kg/24 h) in addition to LEV and PB as part of the oral medical treatment for an intracranial mass lesion. Dog 1 was receiving allopurinol 10 mg/kg/12h due to a diagnosis of leishmaniasis prior to the diagnosis of IE.

### Effect of zonisamide treatment on serum phenobarbital concentrations

In six out of ten dogs (dogs 1, 2, 3, 4, 8, and 9), oral ZN treatment was started with no other ASD dosage change at that time. After a mean of 4.8 weeks (2.5–8 weeks), sPBc was measured. Of these six, four (dogs 1, 2, 3, and 8) showed increased sPBcs at next measurement following the addition of ZN [mean 3.8 weeks (2.5–6 weeks)], despite no change in PB dosage (Table 1). The serum ZN concentration (sZNc) was within the recommended target range of 10–40 mg/L for all four dogs (Table 1). Two out of six dogs (dog 4 and dog 9) did not show an initial increase in sPBc following the addition of ZN.

Of the six dogs, one (dog 4) showed increased sPBc at 37 weeks and 58 weeks following the addition of oral ZN, despite no change in PB dosage (Table 1). In this dog, oral dosage of ZN was increased at 8 and 37 weeks because of an increase in ES frequency (Table 1).

In one dog (dog 9), no increase in sPBc was seen after starting ZN. The peak and trough sPBcs were 32.2 and 31.8 mg/L, respectively, when ZN was started at 7.4 mg/kg/12 h. After 5 weeks, peak and trough sPBcs were 32.3 and 29.1 mg/L, respectively. The sZNc was 11 mg/L. This was the only case in which an increase in sPBc was not noted after starting oral ZN treatment.

Four of ten dogs (dogs 5, 6, 7, and 10) underwent modulation of oral PB dosage when oral ZN treatment was started. Details of these four dogs (dogs 5, 6, 7 and 10) can be found in Table 2.

**Table 2:** Serum PB concentration (sPBc) and the serum ZN concentrations (sZBc) before and after the administrations of zonisamide (ZN) and phenobarbital (PB) at constant dosages.

	Signalment	Diagnosis	PB dosage	ZN dosage	sPBc when receiving PB and ZN (mg/L)	sZNc when receiving PB and ZN (mg/L)	Time between measurements (weeks)	First sPBc after receiving ZN with no increase in PB dosage (mg/L)	First sZNc when receiving PB and ZN with no increase in PB dosage (mg/L)
Dog 5	10-year Corgi	SE	5.2 mg/kg/12 h	7.5 mg/kg/12 h	P: 34.3 T: 34.1 HL: 948.02	18	8	**P: 47** **T: 41.5** HL: 44.85	23
Dog 6	4-year cross breed	IE	5.2 mg/kg/8 h	10 mg/kg/12 h	P: 38.9 T: 36.4 HL: 41.73	22	8	**P: 48.7** **T: 42.2** HL: 19.35	29
Dog 7	7-year FBD	SE	6.4 mg/kg/12 h *reduced to 5.2 mg/kg/12 h following sPBc measured at 44 mg/L	12 mg/kg/12 h	R: 44*	37	4	**P: 49.9** **T: 44.8**	72
Dog 10	5-year cross-breed	IE	4.5 mg/kg	7.5 mg/kg/12 h	P: 32 T: 25 HL: 22.46	22	3	**P: 34** **T: 28.1** HL: 29.01	49

The values in bold represent increases in sPBc despite the dosage of PB not increasing. Dog 7 (*) had the dosage of PB decreased from 6.4 to 5.2 mg/kg/12 h following sPBc measured at 44 mg/L. P, peak sPBc; T, trough sPBc; R, unknown time of day sPBc taken; HL, PB elimination half-life.

In dog 5, peak and trough sPBcs were measured at 34.3 and 34.1 mg/L, respectively, and the sZNc was 18 mg/L, 15 weeks after oral ZN initiation and concomitant modulation of oral PB dosage. The dosage of PB (5.2 mg/kg/12 h) and ZN (7.5 mg/kg/12 h) were kept unchanged and, after 8 weeks, peak and trough sPBcs were measured at 47 and 41.6 mg/L, respectively, and sZNc at 23 mg/L.

In dog 6, the peak and trough sPBcs were 38.9 and 36.4 mg/L, respectively, and sZNc was 22 mg/L, 5 weeks after oral ZN initiation and concomitant modulation of oral PB dosage. Eight weeks later, despite oral PB dosage (5.2 mg/kg/8 h) and oral ZN dosage (12 mg/kg/12 h) being unchanged, the peak and trough sPBcs were increased to 42.2 and 46.7 mg/L, respectively. The sZNc was 29 mg/L.

In dog 7, the oral PB dosage was decreased from 6.4 to 5.2 mg/ kg/12 h because of sPBc of 44 mg/L, 14 weeks after oral ZN initiation and concomitant modulation of oral PB dosage. The sZNc was 37 mg/L. Three weeks later, peak and trough sPBcs were increased to 49.9 and 44.8 mg/L, respectively, despite a decrease in oral PB dosage. The sZNc was 72 mg/L.

In dog 10, the peak and trough sPBcs were 32 and 25 mg/L, respectively, 6 weeks after oral ZN initiation and concomitant modulation of oral PB dosage. Three weeks after oral ZN dosage was increased from 7.5 to 15 mg/kg/12 h, and the dosage of PB remained unchanged at 4.5 mg/kg/12 h, peak and trough sPBcs had increased to 34 and 28.1 mg/L, respectively. The sZNc was 49 mg/L.

### Calculation of phenobarbital elimination half-life

#### Dogs that did not undergo PB modulation at the start of ZN treatment

Phenobarbital elimination half-life could be calculated in five out of ten dogs (dogs 1, 2, 3, 4, and 9) at the beginning and 3–8 weeks after starting ZN (Table 1). Of these, the PB elimination half-life was increased in three dogs (dogs 1, 2, and 3) and decreased in two dogs (dogs 4 and 9).

#### Dogs that underwent PB modulation at The start of ZN treatment

PB elimination half-life could be calculated in three out of ten dogs (dogs 5, 6, and 10) before and 4–8 weeks after dosages of PB were kept consistent (Table 2). Of these, the PB elimination half-life was increased in one dog (dog 10) and decreased in two dogs (dogs 5 and 6).

### Serum triglyceride concentration and bile acid stimulation testing

Of ten 10 dogs, nine showed increased sPBcs following the initiation of oral ZN, and serum triglyceride was measured at the same time in five out of nine dogs and was within normal range in all the cases (<1.6 mmol/L) (17).

In six out of nine dogs (dogs 1, 2, 3, 5, 6 and 10), a BAST was available for assessment. Five of nine dogs (dogs 1, 2, 3, 5, 6) had a BAST performed at the time or after increases in sPBc were noticed, one dog (dog 10) had a BAST 3 weeks prior, one dog (dog 7) had resting bile acids measured, and two dogs (dogs 4 and 8) did not have a BAST or resting bile acids measured at any time period after starting ZN.

For the dogs in which a BAST was performed, one out of six dogs (dog 6) had an abnormal BAST with pre-prandial bile acids of 13 umol/L (reference <10umol/L) and post-prandial bile acids of 140 umol/L (reference <25 umol/L). The trough sPBc was 42.2 mg/L and the sZNc was 29 mg/L. The dosage of PB and ZN were decreased (PB dosage from 5.2 to 3 mg/kg/8 h and ZN dosage from 10 to 5 mg/kg/12 h). After a month, peak and trough sPBcs were 30.6 and 29.5 mg/L, respectively, sZNc was 11 mg/L, and pre- and post-prandial bile acids were within normal reference range.

Dog 7 did not have a BAST performed but did have resting bile acids measured at >140umol/L (reference <10umol/L). In this dog, the previously measured sPBc was 44 mg/L following a recent increase in PB dosage. The dosage of PB was therefore decreased (5.2 to 3 mg/kg/12 h), and 4 weeks later, the peak and trough phenobarbital were 49.9 and 48.8 mg/L, respectively, which was measured at the same time as the resting bile acids. Dog 7 had an intracranial mass lesion and was euthanised shortly afterwards due to increased ES frequency and marked sedation and ataxia.

### Dogs with serum phenobarbital concentrations above reported hepatotoxic concentration (>35 mg/L)

**Figure 1:**
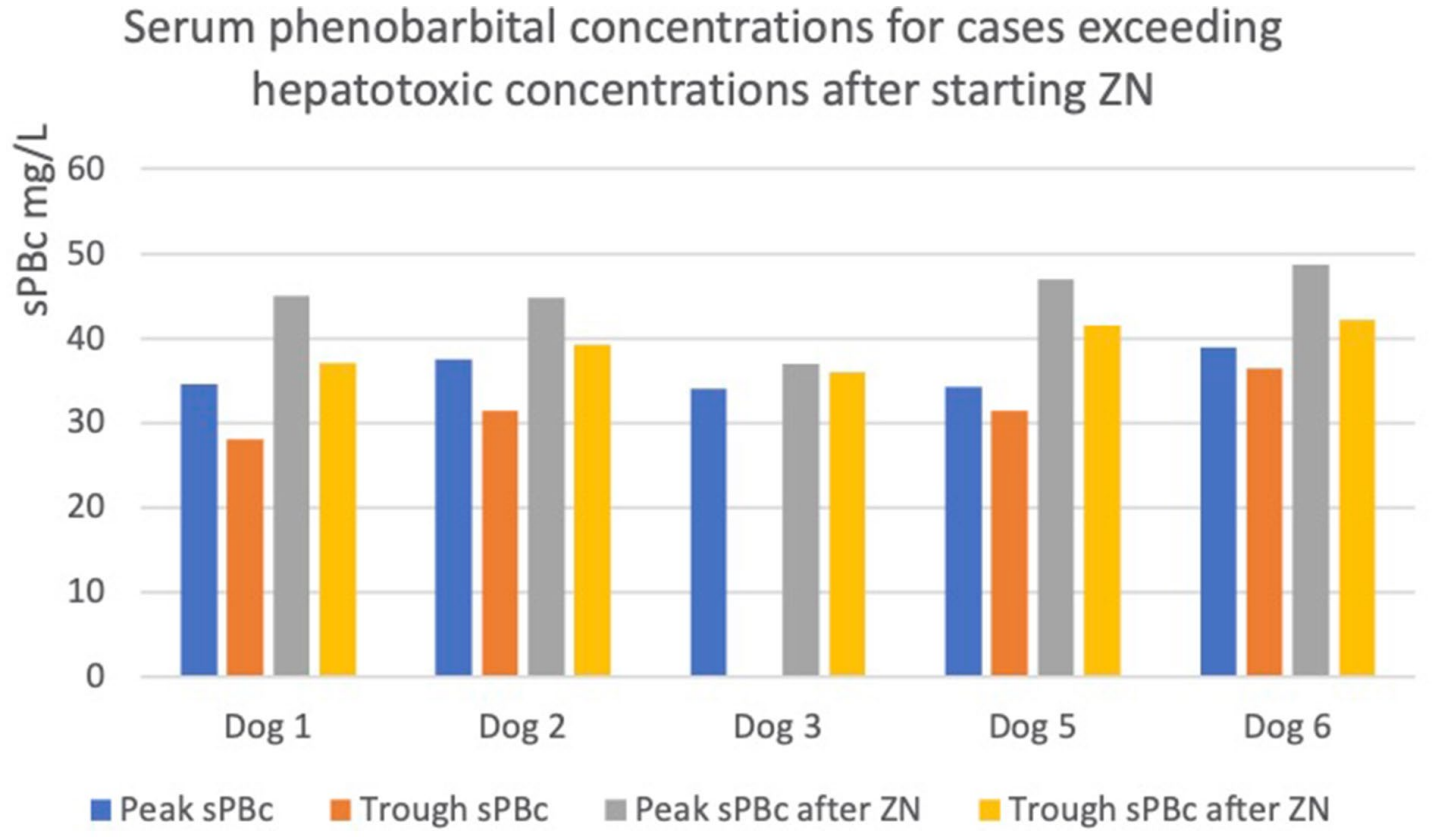
Graph showing increases in peak and trough serum phenobarbital concentrations (sPBc) following administration of zonisamide (ZN) in dogs with sPBc increased to above the reported hepatotoxic concentration, 35mg/L. The dotted line represents 35mg/L.

In five out of ten dogs (dogs 1, 2, 3, 5, and 6), the sPBc was increased to such as extent despite no change in oral PB dosage, leading to a concern of potential hepatoxicity due to trough concentrations being above 35 mg/L (5) (Figure 1).

In particular, in dogs 1 and 2, the trough sPBcs were within the target range before starting ZN and were found to be above the target range when measured 6 and 7 weeks later, respectively (Table 1). Dog 3 initially had an increase in sPBc after starting on ZN (Table 1). Due to poor epileptic seizure control, the dosages of PB and ZN were increased to 4.4 and 12.5 mg/kg/12 h, respectively, and 8 weeks later, the sPBc was 34 mg/L (taken 8 h after the morning dose) and the sZNc was 32 mg/L. The dosages of PB and ZN were unchanged, and peak and trough sPBc 1 year later were 37 and 36 mg/L, respectively, and the sZNc was 48 mg/L. Dogs 5 and 6 are described above.

Following trough sPBcs >35 mg/L, four out of five dogs (1, 2, 5, 6) had the oral PB dosage decreased, and one dog was lost to follow-up (dog 3).

All dogs (dogs 1, 2, 3, 5, and 6) had a BAST performed when sPBcs>35 mg/L, which was normal in four out of five cases. One of five dogs (dog 6) who had a BAST performed had abnormal results (described above).

Additionally, in dog 7, despite a decrease in oral PB dosage (due to sPBc being 44 mg/L), sPBc had risen to peak and trough sPBcs of 49.9 and 44.8 mg/L, respectively, after 4 weeks. Dog 7 had resting bile acids measured, which was abnormal (described above).

### Antiseizure drug treatment: adverse effects

One dog (dog 7) had mild transient ataxia when oral ZN treatment was initially added to the chronic ASD treatment. Both sZNc and sPBc were within the target range. Five out of ten dogs (dogs 1, 2, 5, 6, and 7) experienced generalised ataxia and sedation during treatment. Adverse effects were suspected to be associated with sPBc>35 mg/L in all the five dogs. In four of these five dogs (dogs 1, 2, 5, and 6), all adverse effects improved following a decrease in oral PB dosage. However, in one dog (dog 7), continued sedation and ataxia occurred as well as an increase in ES activity. Dog 7 had an intracranial mass and was therefore euthanised 10 days after a decrease in PB dosage due to continued ES activity and poor quality of life according to its owner.

## Discussion

To the best of our knowledge, this is the first report of the impact that oral ZN treatment could have on the metabolism of PB in dogs with IE and StrE. The metabolism of PB is by the hepatic microsomal enzymatic system, particularly the cytochrome P450 enzyme CYP2C (14). It has been shown that PB is a potent inducer of cytochrome P450 enzymes CYP1A, 2C, 2D, and 3A (14). Over time, chronic PB treatment accelerates its own metabolism, thus reducing its elimination half-life. It is therefore expected that in dogs chronically treated with PB, sPBcs will decrease over time (14, 21). When the PB half-life reduces to less than 20 h, ES control can be improved by the administration of PB every 8 h (18).

Zonisamide is initially started at a dosage of 3–7 mg/kg/12 h as monotherapy, and it has been recommended to increase the dosage to 7–10 mg/kg/12 h when given concurrently with PB in epileptic dogs (3, 9). This is due to the ability of PB to induce hepatic enzymes, including CYP2C and CYP3A, which metabolise ZN in humans (15). In dogs, ZN is metabolised more quickly when PB is administered concurrently, hence a higher dosage of ZN is required when used with PB (12). However, the influence of ZN on PB metabolism has not been reported in dogs. We speculate that the metabolism of PB could be decreased when given alongside ZN due to their shared metabolic pathways.

There are no studies documenting how ZN could affect PB metabolism in humans. An *in vitro* study conducted in human liver microsomes showed that the activity of the cytochrome P450 enzymes CYP2A6, CYP2C9, CYP2C19, and CYP2E1 were reduced in the presence of ZN in an apparent concentration-dependent manner. However, reduction of more the 25% of the enzymatic activity only occurred when the concentrations of ZN were more than four to six times higher than the typical target concentrations (C_max_ of 159 ± 51 μM after a single administration of 8 mg/kg/day of ZN) (22). How the activity of cytochrome P450 enzymes are affected in dogs treated with ZN is unknown. Potentially the administration of ZN reduces the activity of cytochrome P450 enzymes leading to a decreased metabolism of PB, but further studies are required to investigate this. Only half the dogs in this report had an increased PB elimination half-life measured following ZN administration, and therefore, a larger population of dogs is required to investigate the pharmacokinetic interactions.

The recommended target range of ZN for dogs with epilepsy is 10–40 mg/L based on extrapolation from human medicine (5, 7). Interestingly, the two dogs (dogs 4 and 9) that had sZNc measured (<20 mg/L) did not exhibit an increase in sPBcs at initial subsequent measurements. In dog 4, an increase in sPBc was not noted until 37 weeks later when the sZNc had increased to 35 mg/L. In dog 9, no further analysis was available. It could therefore be hypothesised that sPBc could be affected by sZNc in a dose-dependent manner over time.

This case series suggests that sPBcs should be carefully monitored following the addition of ZN, particularly in those dogs that have sPBc at the higher end of the recommended reference range (15–35 mg/L) (5). The three dogs (dogs 3, 5, and 6) that had sPBcs above 30 mg/L and were continued on the same dosages of PB and ZN or had the ZN dosage increased were found to have subsequent sPBcs measured at >35 mg/L. Of the two dogs that had trough sPBc of >30 mg/L before starting ZN, one had a trough sPBc >35 mg/L at next measurement (dog 2). Trough sPBc >35 mg/L increases the risk of hepatotoxicity and indeed one dog (dog 6) with trough sPBc >35 mg/L had an increased bile acid stimulation test, suggestive of liver dysfunction (5, 23). As was shown in dog 6, the effects can be reversible; however, PB-induced hepatoxicity can be fatal if left untreated (6).

Previous studies in dogs treated with ZN found that 10–55% dogs experienced side effects including ataxia, sedation, inappetence, or gastrointestinal signs, which were transient in the majority of cases (7–9). Indeed, dosages as high as 75 mg/kg/24 h appear to be still well tolerated in dogs (24). In the dogs in the study, there were very few side effects with the addition of ZN, with only one dog demonstrating a mild transient ataxia when sPBc and sZNc were within the target ranges. However, generalised sedation and ataxia was noticed in five of six dogs with sPBc >35 mg/L. This finding additionally supports that sPBcs should be carefully monitored following the addition of ZN, as side effects of ASD can markedly affect a dog’s quality of life (25).

All dogs except one (dog 8) were receiving one or more ASMs in addition to PB and ZN. Seven dogs were receiving LEV and two dogs were receiving LEV and KBR. The likelihood of LEV or KBR affecting PB metabolism is low due to the lack of protein binding, hepatic metabolism, and induction of hepatic enzyme (5). Neither LEV nor KBR has been reported to affect the pharmacokinetics of any ASM in dogs or cats, although there are no specific studies about the pharmacokinetics of PB when given concurrently with LEV or KBR in dogs. It has been shown that PB can increase the clearance of LEV when they are given together (5, 26). Based on the available literature, it was assumed unlikely that LEV or KBR administration could have affected the sPBc and therefore the results of this study.

We are aware that this study has several limitations. First, due to its retrospective nature, the time between ASM serum concentration measurements were not standardised. A study looking at set time points for sPBc measurements after starting ZN would be recommended to support our findings. Despite this limitation, reporting this case series of dogs living with a lifelong condition such as IE provides the opportunity to understand the realistic clinical practice.

Liver dysfunction could potentially lead to unexpected increases in sPBcs due to changes in drug metabolism, and not all dogs had a BAST (27). However, five out of six dogs who did have a BAST had normal results, making liver dysfunction causing a decrease in PB metabolism unlikely in these cases. For the one dog with an abnormal BAST, when the dosage of PB was decreased and the sPBcs were within target range, the subsequent BAST was normal. Therefore, it seemed more likely that the increased sPBcs were causing hepatoxicity, leading to the increased BAST, rather than a primary hepatopathy leading to changes in PB metabolism. The one dog with increased resting bile acids was euthanised (due to intra-cranial mass and poor quality of life) shortly after these blood tests and, therefore, a hepatopathy could not be excluded.

Due to the retrospective nature of the study, PB formulation was only available for seven out of ten dogs. Switching PB brand may cause alterations in sPBcs, and so it cannot be excluded that PB brand changes could have influenced the sPBcs in the three dogs whose data were not available (28).

Not all dogs had their serum triglyceride concentrations measured. Fasting samples are usually recommended when measuring sPBc as serum triglyceride concentrations >11.3 mmol/L have been associated with a false elevation in sPBc (17). However, considering that five out of nine dogs had serum triglyceride concentrations measured and they were all within the target range, it is unlikely that hypertriglyceridemia could have affected our results. It has been shown that PB fluctuations can occur throughout the day, although this is only significant when the dosage is more than 10 mg/kg/24 h (21). Although the majority of dogs had peak and trough sPBcs measured (18), in one dog (dog 8), comparisons were made between single samples before and after starting ZN. However, due to the fact that the samples were reportedly taken at the same time of day, and the dog was receiving <10 mg/kg/24 h of PB, it was considered unlikely that the increase in sPBc was due to daily fluctuations and rather a result of the ZN administration.

In conclusion, this is the first study reporting the effect that oral ZNS treatment may have on PB serum concentrations in epileptic dogs. The addition of oral ZN to oral PB chronic treatment might result in significant increases in sPBc, requiring modulation in oral PB dosage, and we suggest that sPBcs should be carefully monitored due to the risk of hepatoxicity. More studies are required to fully establish the pharmacokinetic interactions between PB and ZN in chronically treated dogs.

## Data availability statement

The original contributions presented in the study are included in the article/supplementary material, further inquiries can be directed to the corresponding author.

## Ethics statement

Ethical approval was not required for the studies involving animals in accordance with the local legislation and institutional requirements because it is a retrospective study in which owners consented for the data to be used. Written informed consent was obtained from the owners for the participation of their animals in this study.

## Author contributions

EM: Writing – original draft. OM: Writing – review & editing. AU: Writing – review & editing. FS: Writing – review & editing.
